# Complex skull base brain tumor resection: the role of microvascular doppler in surgical precision and outcomes

**DOI:** 10.3389/fonc.2025.1600980

**Published:** 2025-09-03

**Authors:** Kecheng Shen, Liang Sun, Weiwei Zhai, Lijuan Kong, Jiandong Zhu, Zhengquan Yu, Jiang Wu

**Affiliations:** Department of Neurosurgery, The First Affiliated Hospital of Soochow University, Suzhou, Jiangsu, China

**Keywords:** skull base, brain tumor, microvascular doppler, neurosurgery, neurosurgical outcomes

## Abstract

**Objective:**

To investigate the role of Doppler microvascular ultrasound (MVD) in skull base surgery for the intraoperative assessment and protection of arterial blood flow.

**Methods:**

The clinical data of 56 patients who underwent surgery at the Department of Neurosurgery, First Affiliated Hospital of Soochow University between September 2017 and June 2024 were retrospectively analyzed. All patients underwent skull base tumor resection assisted by intraoperative microvascular Doppler (MVD). The procedures involved complex skull base tumors, including lesions in the sellar region, sphenoid ridge, and cavernous sinus. During surgery, MVD was utilized to monitor blood flow in vessels adjacent to the tumors. The spatial relationship and patency of the vessels and tumors were evaluated in conjunction with preoperative multimodal imaging.

**Results:**

No intraoperative vascular injuries were observed among the 56 patients, confirmed by intraoperative Doppler monitoring and postoperative imaging. All patients had favorable postoperative outcomes, including no new neurological deficits. MVD facilitated precise intraoperative localization and evaluation of blood vessels, which was quantitatively supported by significant increases in contrast-to-noise ratio (CNR) after Doppler application. In two representative cases, the addition of FLOW800 fluorescence imaging provided further CNR improvement, suggesting enhanced visualization and vascular protection. Details of the CNR quantification process are provided in the Methods section.

**Conclusion:**

MVD provides real-time intraoperative information on arterial blood flow during skull base surgeries, enabling surgeons to identify and preserve critical vessels, thereby improving surgical safety, resection accuracy, and patient outcomes. With ongoing technological advancements, the integration of MVD and fluorescence imaging is expected to play an increasingly vital role in complex skull base procedures.

## 1 Introduction

Surgical treatment of skull base tumors is one of the most challenging and complex aspects in neurosurgery. Skull base tumors refer to tumors that occur in the region of the base of the skull, which includes the bottom of the brain and the bone structures and neural vessels that are crucial to the brain. Due to the complexity of the skull base structure and its important anatomical location, surgical treatment of skull base tumors requires highly specialized techniques and experience (1, 2).

Skull base tumors can be benign or malignant. Common types of skull base tumors include meningiomas (3), schwannomas (4, 5), and pituitary tumors (6). Due to the tumor’s proximity to cranial nerves and important blood vessels, symptoms may include headache, visual impairment, hearing loss, facial numbness, and even motor function disorders. For example, the cavernous sinus is an important anatomical structure that contains the internal carotid artery and the third, fourth, fifth, sixth cranial nerves. Benign tumors such as meningiomas, trigeminal neural sheath tumors, and cavernous angioma can occur within or extend into this structure, causing damage to visual function, extraocular movement, facial sensation function, and other CN functions (7–9).

Surgical resection is the standard main treatment to achieve immediate reduction in the mass of large tumors and obtain histopathological diagnosis. In most cases, we can make an accurate diagnosis based on the characteristics of advanced neuroimaging. Although microscope and endoscope skull base techniques have been matured, the surgical intervention for such tumors located deep in the skull base and close to the carotid artery, cranial nerves, visual pathway, and pituitary gland remains challenging. To protect cranial large vessels, the surgeon may need to balance the extent of tumor resection, leading to tumor residue, recurrence, or regrowth (10).

To maximally resect the tumor and minimize damage to cranial nerves and cranial large vessels, neurosurgeons have utilized many techniques aimed at improving brain tumor surgical treatment, including preoperative advanced imaging detection, neuronavigation, and multimodal fusion imaging. However, preoperative tools are limited by surgical manipulation and brain shift (11). This limitation can be overcome by using intraoperative imaging, such as intraoperative MRI, and intraoperative microvascular Doppler (MVD). Intraoperative MR has high costs, operational difficulties, and logistical problems, making it difficult to disseminate. On the other hand, intraoperative microvascular ultrasound has been adopted in other surgeries and can be performed multiple times during surgery (12–14). In recent years, the application of MVD in neurosurgery has received increasing attention. MVD aims to perform a complete intraoperative anatomical and vascular evaluation of brain tumors, adjusting surgical procedures based on findings made during the operation. Additionally, MVD can be easily repeated multiple times, thus maximizing the extent of brain tumor resection (EOR) to preserve the function of intracranial major vessels (15–20).

MVD is a relatively inexpensive, convenient, easy-to-use, and useful technique that has been widely used in the surgery of cerebral aneurysms and arteriovenous malformations. It can help properly place aneurysm clips without significantly prolonging surgical time (21). The MVD probe is the simplest and most reliable method for verifying blood flow velocity. It allows the neurosurgeon to non-invasively immediately assess blood flow velocity, verify the proper placement of aneurysm clips, and maintain sufficient blood flow in adjacent vessels. In this study, we applied MVD to the surgery of skull base tumors to evaluate the blood vessel situation around the tumor in real-time during the operation, allowing the tumor to be resected to the greatest extent possible.

In this study, we retrospectively analyzed 56 patients with brain tumors who underwent surgery assisted by MVD at the Department of Neurosurgery, Suzhou University First Affiliated Hospital from September 2017 to June 2024. All patients were preoperatively evaluated as having anatomically complex tumors with high surgical risks, and were closely related to intracranial vessels. Serious consequences would result from surgical injury to the important vessels encircling or adjacent to the tumor. All patients were protected from vascular injury during surgery with the assistance of MVD, and the vessels were well protected. Therefore, we propose that using intraoperative MVD to detect the course of the vessels can reduce the risk of vascular injury.

## 2 Methods

### 2.1 Patients

In this retrospective study, 56 patients from The First Affiliated Hospital of Soochow University underwent surgical treatment from September 2017 to June 2024. All patients had microvascular Doppler (MVD) assistance during surgery. The surgeries included complex sellar region tumors treated with transcranial and transsphenoidal approaches, medial sphenoid ridge tumors, cavernous sinus tumors, petroclival tumors, middle cranial base and infratemporal fossa tumors, and neck tumors. For these tumors, the internal carotid artery system was the target for intraoperative MVD monitoring. For tumors in the cerebellopontine angle, jugular foramen, foramen magnum, and cerebellum, the vertebrobasilar arterial system was the target for intraoperative MVD monitoring.

Perioperative clinical, surgical, and imaging data were collected. The study included 35 female and 21 male patients, with an age range of 24 to 84 years (mean age 51.61 years). This study was approved by the local ethics committee (S2018087) and informed consent was obtained from all patients. The complete dataset of all 56 patients is presented in **Supplementary Table 1**, including surgical details and pathological results.

### 2.2 Intraoperative microvascular doppler

**Figure 1:**
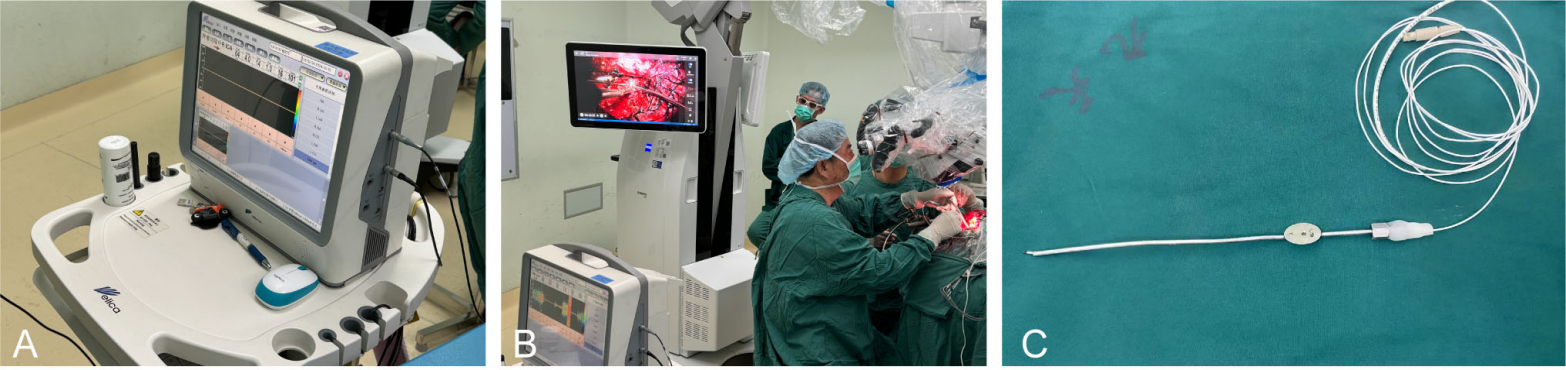
Intraoperative microvascular Doppler used in the surgery. **(A)** German transcranial Doppler device. **(B)** Use MVD for real-time monitoring during surgery. **(C)** The ultrasound probe.

During the procedure, a transcranial Doppler device (EME Companion TC2021-III, Germany) was used for arterial blood flow monitoring. The ultrasound probe had a diameter of 1.5 mm and operated at a frequency of 20 MHz (**Figure 1A**). The operator utilized the MVD to detect blood flow signals within and around the tumor, with a specialized TCD physician observing and recording the flow spectrum morphology and audio signals. Blood flow parameters were analyzed to assess the trajectory and distribution of arteries surrounding and enveloped by the tumor in real time (**Figure 1B**). When using the MVD probe, we combined it with a metal extractor to ensure flexibility and stability during use (**Figure 1C**).

### 2.3 Neuroimaging

All patients underwent preoperative computed tomography (CT) and magnetic resonance imaging (MRI) to evaluate tumor characteristics and plan the surgical approach. Multimodal image fusion was used to assess the spatial relationship between the tumor and surrounding vessels. Tumor volume was measured using T1-weighted gadolinium-enhanced MRI with iPlan Cranial software (BrainLab, Germany).

Gross total resection (GTR) was defined as the complete removal of the tumor, confirmed intraoperatively and quantitatively verified by volumetric analysis on contrast-enhanced postoperative MRI. The extent of resection (EOR) was calculated as:


EOR=[(V_pre−V_post)/V_pre]×100%

where V_pre and V_post represent the tumor volume before and after surgery, respectively. Tumor segmentation and volumetric calculations were performed using iPlan Cranial software (BrainLab, Germany), with GTR defined as EOR = 100%. Additionally, postoperative computed tomography angiography (CTA) was performed to assess the presence of any vascular complications, including vessel occlusion, stenosis, or flow interruption. Postoperative intracranial hemorrhage was monitored by routine head CT scans performed within 24 – 48 hours after surgery. Patency of major arteries was confirmed by the visualization of uninterrupted contrast filling in the internal carotid artery (ICA), middle cerebral artery (MCA), and other relevant vessels in the operative field. This imaging evaluation was independently reviewed by two experienced neuroradiologists.

### 2.4 Surgical equipment

An endoscope (Karl Storz, Germany) and a microscope (Pentero; Carl Zeiss, Germany) were used in the integrated operating room. The endoscopic and microscopic views were displayed simultaneously on high-definition screens visible to the entire surgical team (including the surgeon, assistant surgeons, and assisting nurses).

Successful detection by MVD was defined as the presence of a detectable Doppler signal from major intracranial arteries (e.g., ICA, MCA, BA) intraoperatively. Detection success rate was calculated as:


(number of patients with detectable Doppler signal/total patients assessed) 

In addition to MVD, intraoperative neurophysiological monitoring including brainstem auditory evoked potentials (BAEPs) and motor evoked potentials (MEPs) was applied in selected cases, particularly when operating in proximity to brainstem or cranial nerve structures. These modalities provided real-time intraoperative feedback on functional integrity, operated by trained neurophysiological staff, particularly during brainstem-adjacent procedures. Since these data were not systematically recorded for all patients, they were not included in the **Supplementary Table 1**.

### 2.5 Quantitative image analysis (CNR computation)

To quantitatively assess intraoperative vessel visualization, contrast-to-noise ratio (CNR) analysis was conducted in five representative patients (Cases 24, 41, 55, 56, and 37), who were selected based on the availability of complete and high-quality intraoperative imaging across multiple stages (before and after MVD, and MVD + FLOW800 when available). Intraoperative images were converted to 8-bit grayscale using ImageJ (NIH, USA). Rectangular regions of interest (ROIs) were manually drawn over the target blood vessels and adjacent background tissue for each selected image. μ_v and μ_b represent the mean grayscale intensity values of the vessel and background regions, respectively, as described in **Supplementary Table 2**. The standard deviation of background pixel intensity (σ_b) was also calculated. CNR was defined as:


$$\text{CNR }=\;\left(\text{μ}\_\text{v }–\text{ μ}\_\text{b}\right)/\text{σ}\_\text{b}$$

Measurements were performed before and after MVD in all five cases. Additionally, in Cases 37 and 56, separate images were acquired using MVD and FLOW800 (ICG) at corresponding surgical stages. These images were analyzed independently to compare the vessel-to-background CNR values between modalities. While the two imaging modalities were used in conjunction intraoperatively, CNR values were computed separately for MVD and FLOW800 images without merging or overlay. For dual-modality comparison, MVD and FLOW800 images were analyzed independently, with ROIs drawn separately on each modality to calculate CNR without image overlay. Full grayscale values and computed CNR values are listed in **Supplementary Table 2**.

Representative grayscale images with manually defined ROIs for vessel and background regions are provided in **Supplementary Figures 1A–L**.

## 3 Results

### 3.1 Study population

**Table 1** summarizes the clinical characteristics of 56 patients whose arterial conditions were assessed using MVD intraoperatively. Among the 56 patients, the detection success rate of MVD was 87.63%. According to the postoperative MRI, the GTR rate of the patients was 91.07%. After the operation, we used CTA for vascular assessment, and the blood vessels of all patients were well protected. During the follow-up period, no radiologically confirmed tumor recurrence was observed. For patients who did not undergo gross total resection, adjuvant stereotactic radiosurgery (e.g., Gamma Knife) was administered, which may have contributed to recurrence prevention.

**Table 1:** Summary of clinical and surgical outcomes grouped by tumor location (n = 56).

Tumor location	n	Mean age (years)	Mean tumor size (cm³)	MVD detection rate (%)	GTR rate (%)	Vascular protection rate (%)
Sella region	24	48.3	2.6 ± 0.4	91.7	95.8	100
Cavernous sinus	6	51.7	3.1 ± 0.5	100	83.3	100
Sphenoid crest	5	64.2	2.9 ± 0.3	80	80	100
Parasellar region	3	60.7	2.7 ± 0.5	100	100	100
CPA	1	73	3.5	100	100	100
Neck/Skull base	8	49.5	3.0 ± 0.6	75	87.5	100
Other	9	45.1	2.8 ± 0.3	66.7	88.9	100
Total	56	51.6	2.9 ± 0.4	87.63	91.07	100

MVD, microvascular Doppler; GTR, gross total resection.

**Figure 2:**
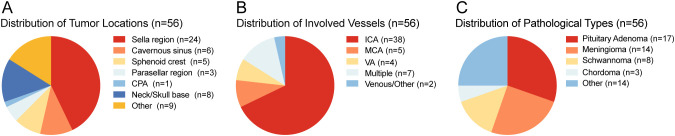
**(A)** Distribution of tumor locations. **(B)** Distribution of involved vessels. **(C)** Distribution of pathological types. ICA, internal carotid artery; MCA, middle cerebral artery; VA, vertebral artery; CPA, Cerebellopontine Angle.

Among them, we analyzed the tumor location, pathology and involved blood vessels of the patients. Among the 56 patients, the largest number was in Sella region, with 24 cases (**Figure 2A**). Internal carotid artery (ICA) is the most affected blood vessel, with a total of 38 patients’ tumors involving ICA (**Figure 2B**). Based on postoperative histopathological examination, the most common type was pituitary adenoma, with a total of 17 cases, followed by meningioma, with 13 cases (**Figure 2C**). During the study period, no patients died of vascular problems. Postoperative computed tomography angiography (CTA) demonstrated that all major intracranial arteries were well preserved without evidence of stenosis, occlusion, or flow disruption, no delayed vascular injury and no postoperative intracranial hemorrhage.

### 3.2 Application of MVD assistance in craniotomy for the resection of complex skull base tumors

For patients with skull base tumors encasing multiple blood vessels, where preoperative CTA and MRA demonstrated patent vessels with unobstructed blood flow, it is challenging to distinguish the tumor from the blood vessels under the microscope during surgery. Preoperatively, we use multimodal fusion to reconstruct and integrate the 3D images of blood vessels and tumors, clarifying their relative positions and the status of blood flow.

**Figure 3:**
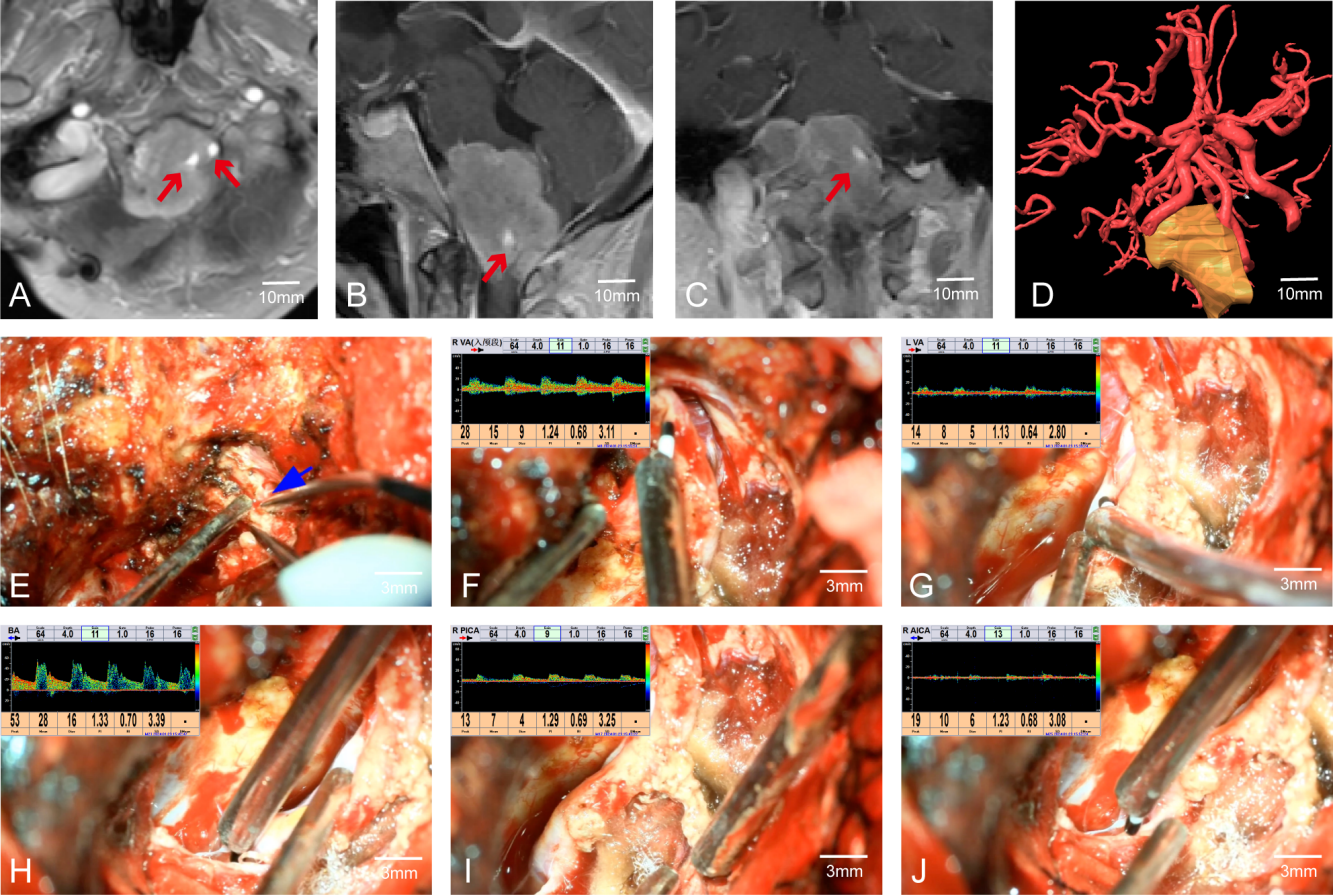
Case 41. **(A–C)** MRA indicates that the tumor envelopes the bilateral vertebral arteries. **(D)** Preoperative multimodal fusion. **(E)** The location of the vertebral artery before the operation. **(F–J)** Postoperatively, MVD is used to confirm the location and patency of the vertebral artery and its branch vessels.

In Case 41, preoperative MRI indicated a tumor in the cerebellopontine angle region, causing severe compression of the medulla (**Figures 3A–C**). The patient had difficulty walking, accompanied by posterior cranial nerve symptoms such as choking on water, difficulty swallowing, and breathing issues, with bilateral lower limb muscle strength at grade II and bilateral upper limb muscle strength at grade IV. MRA suggested the tumor encased both vertebral arteries (VAs). Through multimodal fusion, we found the tumor encased the right VA and was closely related to the left VA (**Figure 3D**). This case represents a typical and challenging scenario of skull base tumors with vascular encasement.

During craniotomy, it was difficult to distinguish the tumor from the blood vessels (**Figure 3E**). We used MVD to detect and identify the courses of both VAs, confirming the relationship between the VAs and the tumor as shown preoperatively by multimodal fusion. This cross-validation allowed us to protect the blood vessels well. Without MVD assistance, safely dissecting the tumor from the encased right VA would have been extremely challenging and posed a high risk of vascular injury. Post-tumor resection, re-detection of the VAs showed good pulsatile blood flow in the VAs and surrounding branches (**Figures 3F–J**). The patient had a good prognosis with significant relief of medulla compression.

### 3.3 Application of MVD assistance in endoscopic resection of complex pituitary tumors

**Figure 4:**
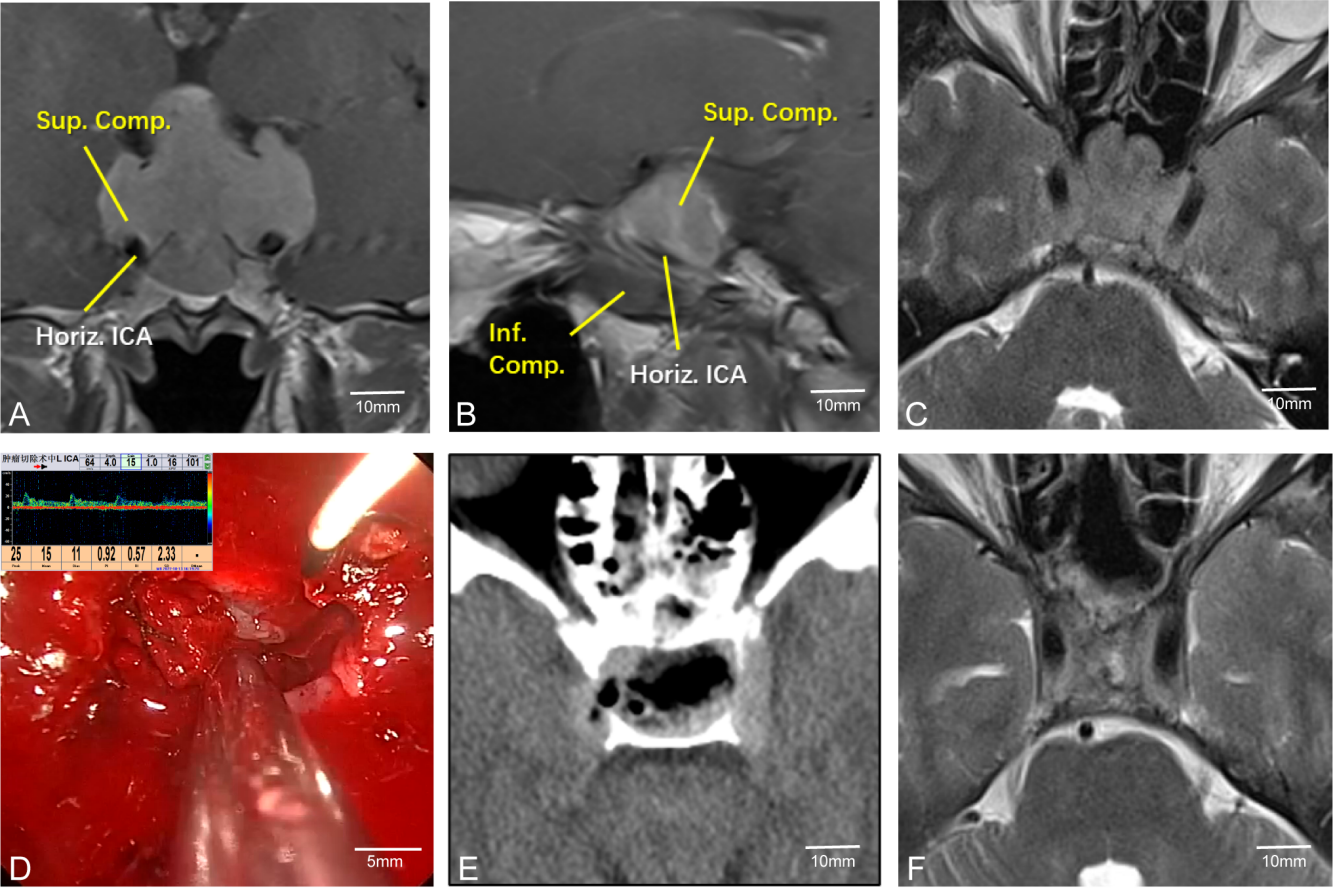
Case 24. Endoscopic resection of complex pituitary adenomas assisted by MVD. **(A–C)** Preoperative MR Showed that the left ICA was surrounded by the tumor. **(D)** Intraoperative determination of the position of the left internal carotid artery and the direction of blood flow. **(E, F)** Postoperative CT and MR demonstrated that the tumor was resected satisfactorily and the blood flow of the internal carotid artery remained unobstructed.

Pituitary tumors with high Knosp grades often increase the difficulty of surgery due to encasement of the C2 or C4 segment of the internal carotid artery (ICA). Intraoperative maneuvers must be extremely careful to avoid damaging the ICA, allowing the removal of tumors hidden in the posterior angle of the ICA and improving surgical outcomes. Therefore, accurately determining the position of the ICA during surgery is crucial. For instance, in Case 24, the left ICA was completely encased by the tumor (**Figures 4A–C**). During surgery, MVD was used to confirm the position of the ICA (**Figure 4D**). The tumor was completely resected while preserving the integrity of the blood vessel (**Figures 4E, F**).

### 3.4 Application of combined FLOW800 fluorescence imaging and MVD in complex skull base tumors

**Figure 5:**
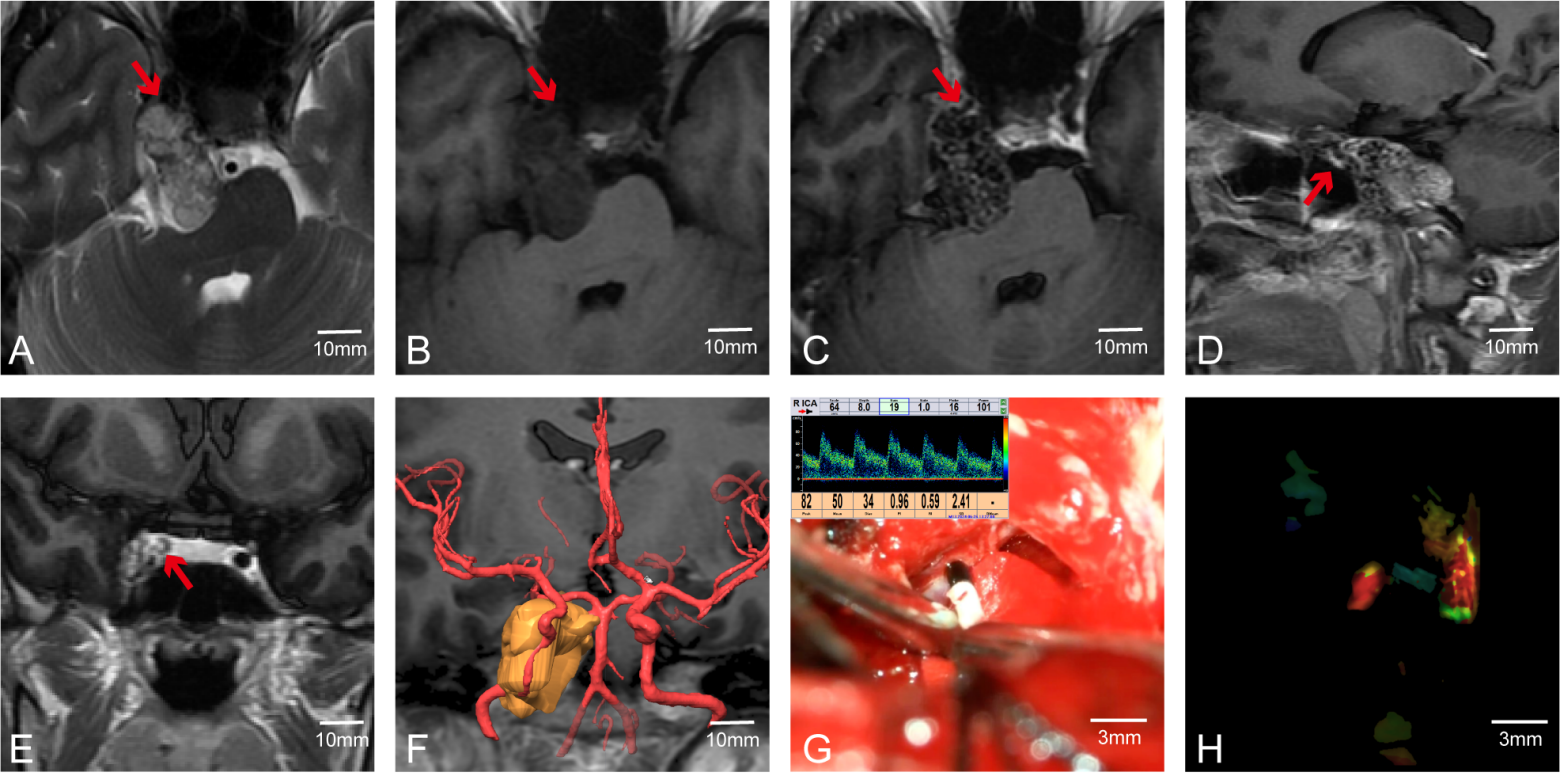
Case 56. The combined use of MVD and FLOW800 in skull base tumor surgery. **(A–E)**Preoperative MR showed that the right ICA was anterior to the tumor. **(F)** Preoperative multimodal fusion. **(G, H)** The internal carotid artery was detected during the operation and compared with FLOW800.

In addition to using MVD to determine the position (anatomical location) and course (spatial trajectory and direction) of arteries—particularly important in skull base tumors where vessels like the internal carotid artery (ICA) may be displaced or encased—we also employed FLOW800 fluorescence imaging during surgery. After injecting indocyanine green, FLOW800 technology provides fluorescence imaging of blood vessels to confirm their positions. For instance, in Case 56, the tumor was located in the right cavernous sinus and clivus (**Figures 5A–E**). Preoperative multimodal fusion showed the ICA located anteroinferior to the tumor and encased by it (**Figure 5F**). During surgery, we used MVD and FLOW800 to mutually confirm the ICA position (**Figures 5G, H**). The tumor was completely resected while preserving the blood vessels. MVD can show the direction and flow of blood, while FLOW800 can display the vessel diameter. The combined use of both methods offers better protection of blood vessels.

### 3.5 Limitations of MVD in skull base surgery

**Figure 6:**
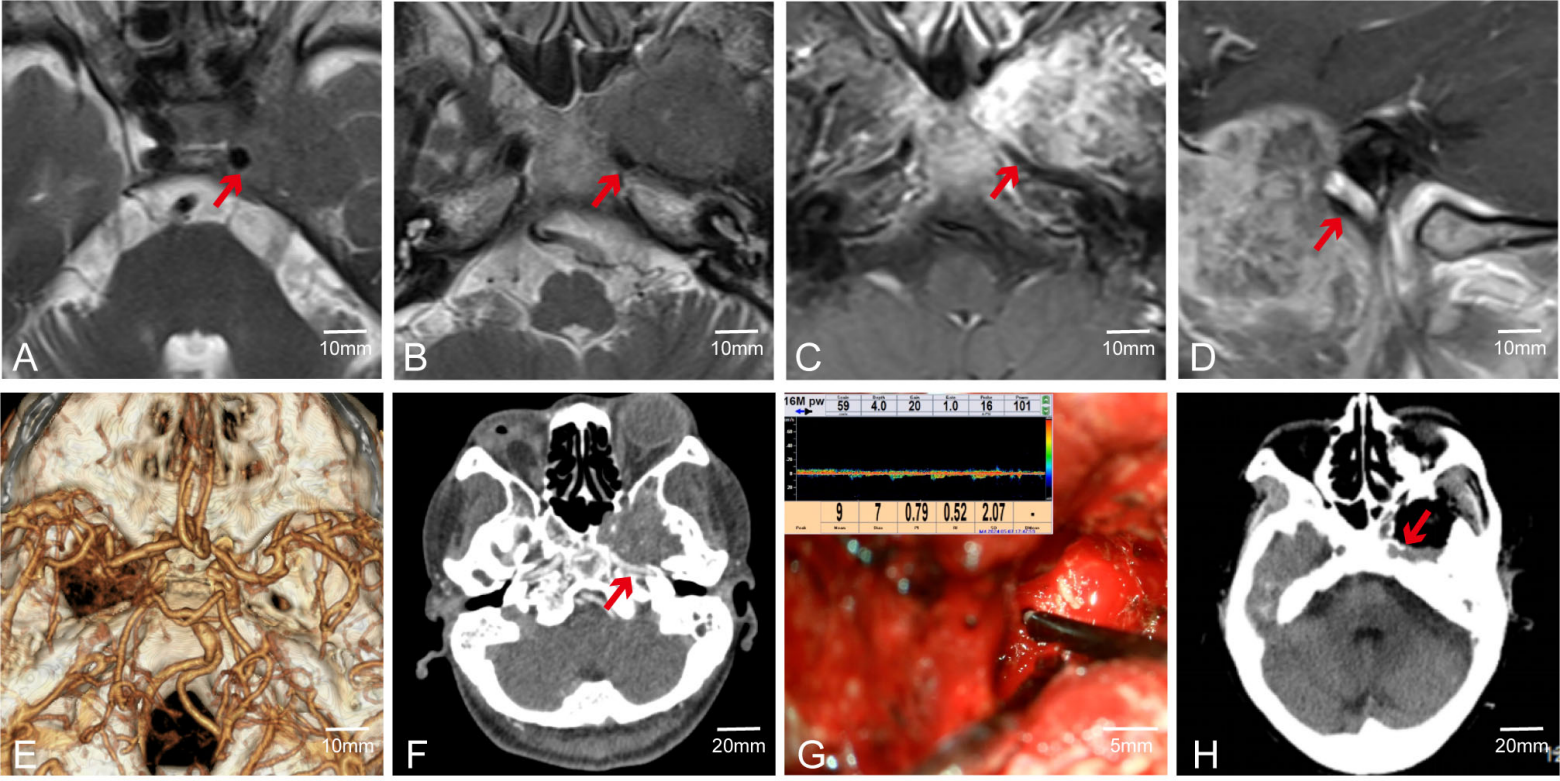
Case 55. MVD combined with CTA was used to evaluate the vascular condition of patients. **(A–D)** Preoperative MR showed that the ICA was closely related to the tumor. **(E, F)** Preoperative CTA. **(G)** Intraoperative fail to detect internal carotid artery. **(H)** Postoperative CT showed ICA lateral has a thin layer of bone.

Although MVD can often assist surgeons in accurately locating major blood vessels during surgery, its effectiveness may be limited when there are structures such as bone or tumor capsules between the probe and the blood vessels. For instance, in Case 55, preoperative CTA was conducted to clarify the spatial adjacency and positional correlation between the tumor, surrounding major blood vessels, and skull base bone structures. Preoperative MRI indicated that the tumor was in close spatial proximity to the internal carotid artery; however, MRI could not adequately assess the integrity of the adjacent bone structures (**Figures 6A–D**). Preoperative CTA suggested the tumor involved the ICA segment at the foramen lacerum and the terminal segment of the petrous bone (**Figures 6E, F**). Post-complete tumor resection, MVD was used to detect the course of the ICA, but no ICA blood flow signal was detected around the tumor (**Figure 6G**). Postoperative CT indicated a thin layer of bone between the ICA and the tumor, with partial destruction of the ICA bony canal (**Figure 6H**). This demonstrates that MVD has weak tissue penetration, and when the probe is not in direct contact with the vessel wall, it may not be able to detect the vessel. In such cases, we rely on electrophysiological monitoring to ensure the blood vessels are not damaged.

### 3.6 CNR-based evaluation of vessel visualization

CNR values were analyzed in five representative cases (Cases 24, 41, 55, 56, and 37). The average CNR increased from 0.87 ± 0.40 before MVD to 2.83 ± 0.37 after MVD(p = 0.0027, paired t-test). Detailed grayscale data and computed CNR values are shown in **Supplementary Table 2**. **Supplementary Figure 1** shows grayscale images, ROI placements, and imaging stages corresponding to the CNR measurements.

An additional patient (Case 37) with complete intraoperative imaging at all three stages (before MVD, after MVD, and during FLOW800 imaging) was included to enable comparative evaluation of different intraoperative imaging modalities, in accordance with reviewer suggestions.

**Figure 7:**
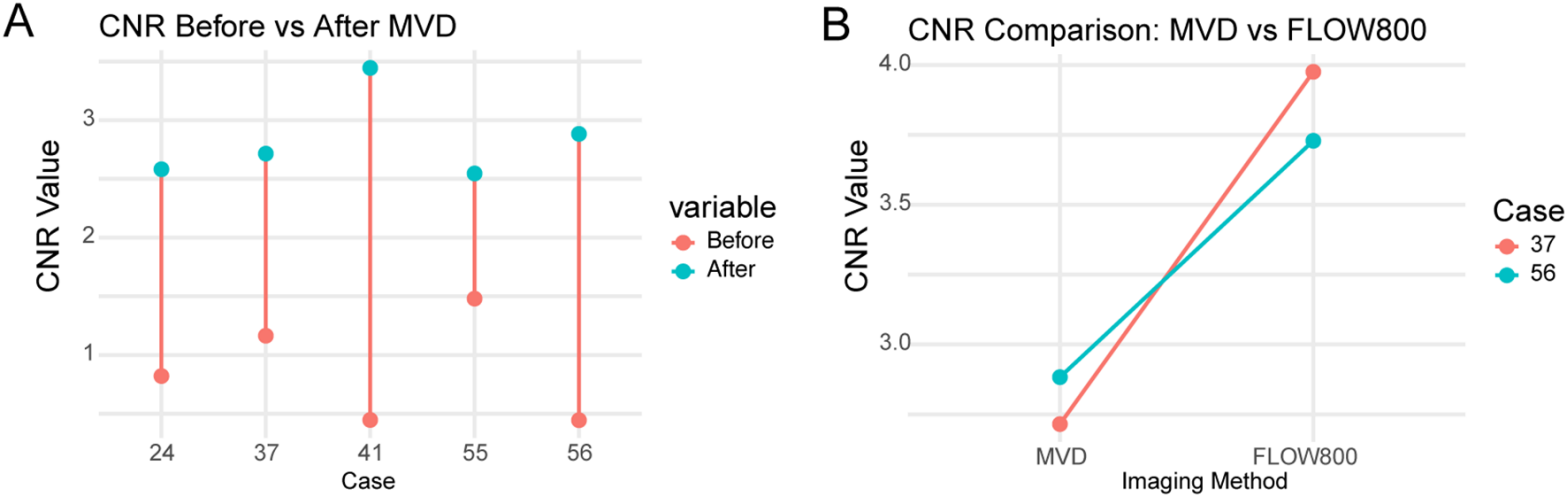
Paired contrast-to-noise ratio (CNR) comparisons in five patients undergoing skull base tumor resection. **(A)** CNR values before and after intraoperative microvascular Doppler (MVD) application in Cases 24, 41, 55, 56, and 37, demonstrating consistent improvement following Doppler-based vessel identification. **(B)** CNR values from MVD and FLOW800 images in Cases 37 and 56, showing higher vessel-to-background contrast with fluorescence-based perfusion imaging. Each line connects the same patient. CNRs were calculated independently from grayscale images acquired using each modality. No image merging or overlay was performed.

In these two cases (Cases 37 and 56), FLOW800 imaging yielded higher CNR values than MVD alone. The average CNR increased from 2.80 (after MVD) to 3.85 (FLOW800), with an average ΔCNR of +1.05, suggesting improved vessel-background contrast with fluorescence-based imaging. These results are illustrated in **Figure 7**.

As only two cases underwent both MVD and FLOW800 imaging, the observed improvements in CNR are preliminary and should be interpreted with caution.

## 4 Discussion

Skull base surgery is a complex and high-risk procedure involving the protection of critical structures such as the brainstem, cranial nerves, and major vessels (21). Intraoperative blood flow monitoring and assessment are crucial for surgical success and patient prognosis. Precise anatomical manipulation is fundamental for arterial protection in skull base surgery. Surgeons need a deep understanding of skull base anatomy, particularly the course and branching of key vessels like the middle cerebral artery and posterior communicating artery. Doppler microvascular ultrasound technology is increasingly used in skull base surgery, providing significant support for intraoperative arterial protection and blood flow assessment (22–24).

Our retrospective analysis of patients undergoing tumor resection with MVD assistance from September 2017 to May 2024 at The First Affiliated Hospital of Soochow University showed no vascular injuries, complete tumor resection, and favorable postoperative outcomes. MVD enables accurate intraoperative localization and course determination of arteries, ensuring complete tumor resection without vascular damage and improving patient prognosis.

Doppler microvascular ultrasound uses the Doppler effect to assess blood flow velocity and direction by detecting frequency changes in reflected ultrasound waves. This technology provides real-time blood flow information during surgery, helping surgeons determine the functional status and location of vessels. In skull base surgery, Doppler microvascular ultrasound is widely used to: 1) assess vessel patency, ensuring critical arteries are not damaged or obstructed during surgery; 2) monitor blood flow changes, quickly identifying potential intraoperative thrombosis, stenosis, or flow interruptions; and 3) evaluate postoperative blood flow restoration, assessing blood flow recovery after vessel anastomosis or reconstruction (24, 25).

For instance, in surgeries for cavernous sinus meningiomas encasing the ICA and high Knosp grade pituitary tumors, MVD helps intraoperatively determine the ICA’s location, assess vessel patency, and confirm the vessel’s position and blood flow recovery post-tumor resection. This ensures vessel patency post-surgery, avoiding intraoperative vascular injuries. Preoperatively, BrainLab multimodal fusion technology reconstructs the relative positions of the tumor and vessels on imaging. Intraoperatively, navigation combined with MVD confirms these findings, demonstrating MVD’s effectiveness in displaying vessel locations, avoiding brain shifts caused by pressure changes and surgical manipulations.

Compared to MVD, FLOW800 technology is widely used in skull base surgery for vascular assessment. FLOW800 fluorescence imaging uses fluorescent agents (such as indocyanine green, ICG) in blood vessels, visualized through a special light source. Intraoperative microscopes and camera systems capture fluorescence signals, generating blood flow images. FLOW800 offers high-resolution, real-time dynamic monitoring and non-invasive evaluation, providing clear vascular structure and blood flow information intraoperatively. It enables dynamic blood flow observation, aiding in surgical evaluation. Compared to traditional angiography, fluorescence angiography causes less tissue damage and has high safety (26–29).

However, previous studies indicate that FLOW800 fluorescence angiography has limitations such as poor penetration and suboptimal deep lesion imaging (30). Kato Y et al. reached similar conclusions, stating that FLOW800 is a reliable and useful adjunct to microscope-integrated color ICG video angiography, though limited for deep AVMs (31). Deep lesions require clear and complete exposure before fluorescence analysis. Additionally, there is a risk of allergic reactions to the fluorescent agent.

Compared with FLOW800, MVD is simple to operate and has strong repeatability and accurate detection of deep blood vessels. Compared with ordinary Doppler ultrasound, under the detection of professional ultrasound doctors, MVD can provide real-time vascular information during the operation, including blood flow direction, speed and whether there is plaque formation, and no additional contrast agent is required. However, we can combine FLOW800 and MVD during the operation, which can provide more accurate vascular and blood flow information, improve the safety and accuracy of the operation, and improve the protection of blood vessels.

Quantitative analysis confirmed that intraoperative microvascular Doppler (MVD) significantly enhances the visualization of blood vessels in skull base surgery. The increase in CNR observed across five patients provides objective validation of the subjective improvements noted during surgery.

In two cases (Cases 37 and 56) where both MVD and FLOW800 imaging were available, the CNR values obtained from FLOW800 images were higher than those obtained from MVD images. This suggests that fluorescence-based perfusion imaging may offer superior vessel-to-background contrast compared to Doppler-based structural imaging in certain scenarios.

However, since CNR values were computed independently from separate grayscale images acquired using each modality, this finding reflects a relative comparison between imaging methods rather than a synergistic effect of combined imaging. While Case 37 was newly included for this analysis, it was selected from the same cohort based on imaging completeness.

These results are based on a small sample and should be interpreted cautiously. Further prospective studies are warranted to validate these preliminary findings and investigate the complementary value of combining structural and perfusion-based intraoperative imaging techniques. Although FLOW800 imaging showed higher CNR values than MVD in two patients, this finding is based on a limited sample and lacks statistical power. Future studies with larger cohorts are planned to further evaluate the comparative performance and potential complementary use of MVD and FLOW800 during skull base surgery.

However, in endoscopic surgery, MVD can directly detect blood vessels in deep structures under direct endoscopic view, which FLOW800 cannot do. But the defect of MVD is that the penetrability of ultrasound waves is not as good as that of fluorescence. In the case of structural barriers, MVD cannot accurately detect blood vessels. When neither MVD nor FLOW800 can accurately detect blood flow, we also used neurophysiological monitoring during the operation to monitor the condition of blood vessels in real time.

A control group without intraoperative MVD was not available for this retrospective cohort. Future prospective studies including non-MVD-assisted surgeries will be valuable for more robust comparisons of surgical outcomes and complication rates.

Long-term follow-up blood flow assessment is significant for predicting patients’ neurological recovery and quality of life. Regular Doppler ultrasound examinations monitor vascular health and detect potential issues.

## 5 Conclusion

The application of Doppler microvascular ultrasound (MVD) in skull base surgery provides a valuable and effective tool for real-time monitoring and assessment of arterial blood flow. Our study demonstrated that MVD not only assists in precise vessel localization and protection but also objectively enhances intraoperative vessel visualization, as shown by a significant increase in contrast-to-noise ratio (CNR) following its use.

Furthermore, the combination of MVD with FLOW800 fluorescence imaging resulted in an additional improvement in vessel contrast, supporting the potential for synergistic imaging benefits. These findings highlight the clinical value of integrating structural and perfusion-based modalities to optimize surgical safety and outcomes.

As imaging technologies continue to evolve, the complementary use of intraoperative Doppler and fluorescence-guided techniques is expected to play an increasingly important role in complex skull base procedures, facilitating safer tumor resections and better patient prognoses.

## Data Availability

The original contributions presented in the study are included in the article/**Supplementary Material**. Further inquiries can be directed to the corresponding authors.
