# Supplementary material for: Complex skull base brain tumor resection: the role of microvascular doppler in surgical precision and outcomes
Source: Front Oncol. 2025 Sep 3;15:1600980. doi: 10.3389/fonc.2025.1600980 (PMC12441036; doi:10.3389/fonc.2025.1600980)
Supplement: Supplementary file 1 [file DataSheet1.docx]

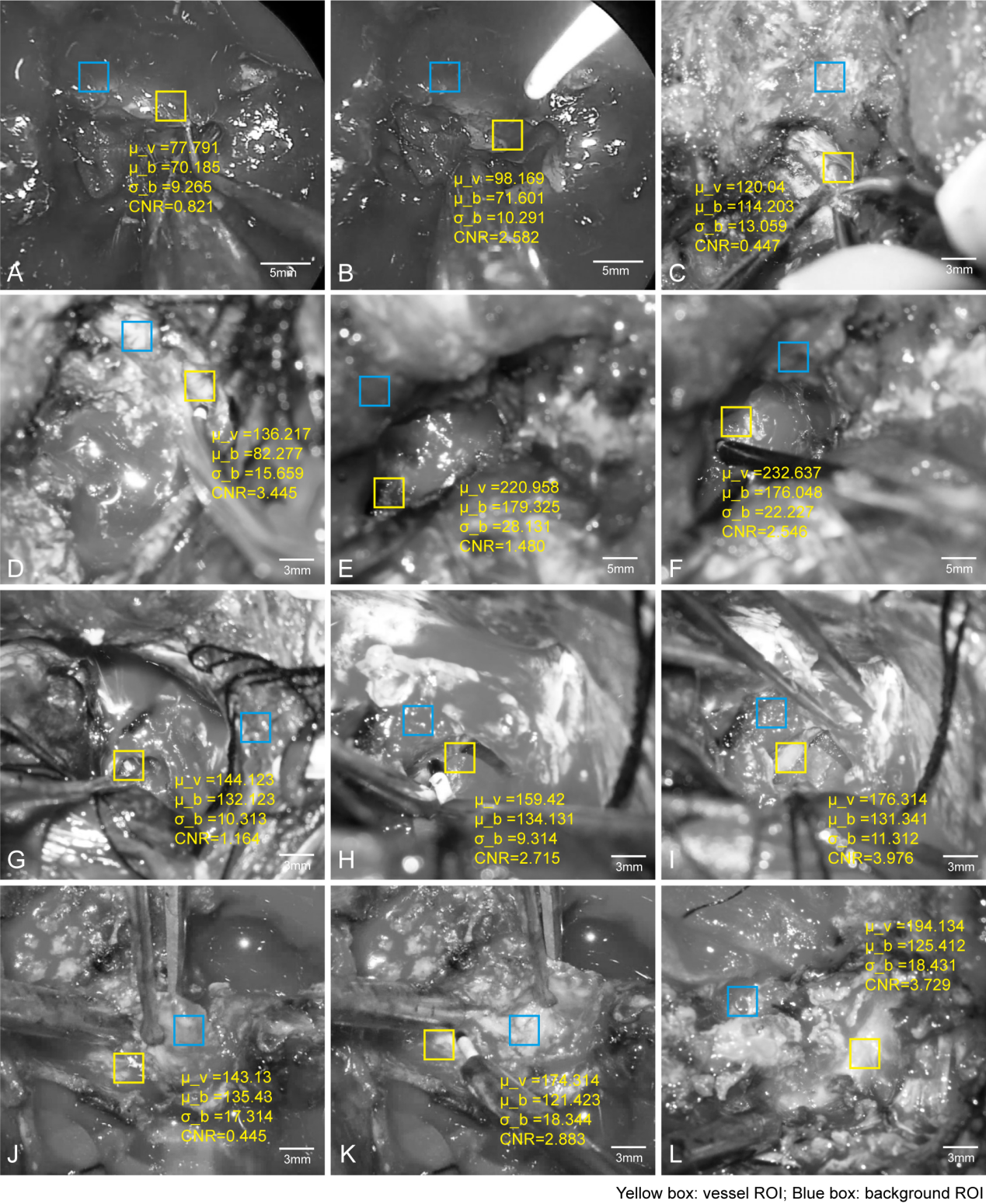

Supplementary Figure 1.

Representative intraoperative grayscale images from five patients (Cases 24, 41, 55, 56, and 37), shown across different imaging stages: before MVD, after MVD, and after MVD combined with FLOW800 (ICG), when available. Panels A–L correspond to the image stages listed in Supplementary Table 2 and are arranged in order. A–B: Case 24 – before and after MVD. C–D: Case 41 – before and after MVD. E–F: Case 55 – before and after MVD. G–I: Case 56 – before MVD, after MVD, and after MVD + FLOW800. J–L: Case 37 – before MVD, after MVD, and after MVD + FLOW800.

For each image, regions of interest (ROIs) were manually selected over the target blood vessel (yellow box) and the adjacent background tissue (blue box). The mean grayscale intensity values (μ_v and μ_b) and background standard deviation (σ_b) were used to calculate the contrast-to-noise ratio (CNR) as follows:

CNR = (μ_v – μ_b) / σ_b.
