# Supplementary material for: Complex skull base brain tumor resection: the role of microvascular doppler in surgical precision and outcomes
Source: Front Oncol. 2025 Sep 3;15:1600980. doi: 10.3389/fonc.2025.1600980 (PMC12441036; doi:10.3389/fonc.2025.1600980)
Supplement: Supplementary file 2 [file Table1.docx]

Supplementary Table 1. Full list of 56 patients undergoing skull base tumor resection with intraoperative Microvascular Doppler (MVD) monitoring.

| Case NO. | Age | Gender | Location | A/V | MVD Detection | Protection | Pathology |
| --- | --- | --- | --- | --- | --- | --- | --- |
| 1 | 67 | Male | Sphenoid crest | ICA | NO | YES | Meningioma |
| 2 | 31 | Male | Infratemporal fossa | ICA | NO | YES | Giant Cell Granuloma |
| 3 | 49 | Female | sella region | ICA | YES | YES | Pituitary Adenoma |
| 4 | 32 | Female | Temporal lobe base | ICA | YES | YES | Hemangiopericytoma |
| 5 | 51 | Female | Parasellar | ICA | YES | YES | Meningioma |
| 6 | 64 | Female | sella region | ICA | YES | YES | Pituitary Adenoma |
| 7 | 56 | Male | cerebellum | Venous sinuses | YES | YES | Diffuse Large B-Cell Lymphoma |
| 8 | 39 | Female | sella region | ICA | YES | YES | Pituitary Chromophobe Adenoma |
| 9 | 30 | Female | Neck | MCA、ICA | YES | YES | Schwannoma |
| 10 | 39 | Female | sella region | ICA | YES | YES | Pituitary Adenoma |
| 11 | 25 | Female | sella region | ICA | YES | YES | Cystic Mature Teratoma |
| 12 | 59 | Female | sella region | ICA | YES | YES | Chondroma |
| 13 | 49 | Male | Occipital | VA | YES | YES | Schwannoma |
| 14 | 59 | Female | sella region | ICA | YES | YES | Pituitary Adenoma |
| 15 | 27 | Male | sella region | ICA | NO | YES | NA |
| 16 | 48 | Female | sella region | ICA | YES | YES | Pituitary Adenoma |
| 17 | 78 | Male | N/A | MCA | YES | YES | Metastatic Pancreatic Adenocarcinoma |
| 18 | 57 | Female | Cavernous sinus | ICA | YES | YES | Solitary Fibrous Tumor (SFT) |
| 19 | 68 | Female | sella region | ICA | YES | YES | Pituitary Adenoma |
| 20 | 56 | Female | sella region | ICA | YES | YES | Diffuse Large B-Cell Lymphoma |
| 21 | 58 | Male | sella region | ICA | YES | YES | Pituitary Adenoma |
| 22 | 49 | Male | sella region | ICA | YES | YES | Meningioma |
| 23 | 58 | Female | sella region | ICA | YES | YES | Pituitary Adenoma |
| 24 | 29 | Male | sella region | ICA | YES | YES | Pituitary Adenoma |
| 25 | 54 | Male | Skull base | ICA | NO | YES | Chordoma |
| 26 | 39 | Male | sella region | ICA | YES | YES | Pituitary Adenoma |
| 27 | 63 | Female | Skull base | VA | YES | YES | Meningioma |
| 28 | 61 | Female | Cavernous sinus | ICA | YES | YES | Meningioma |
| 29 | 41 | Male | sella region | MCA、ICA、ACA、PCA | YES | YES | Meningioma |
| 30 | 54 | Female | Sphenoid crest | ICA、MCA、ACA | YES | YES | Meningioma |
| 31 | 86 | Male | Skull base | ICA | YES | YES | Atypical Meningioma |
| 32 | 43 | Male | sella region | ICA | YES | YES | Invasive Pituitary Adenoma |
| 33 | 56 | Female | Neck | MCA、ICA | YES | YES | Schwannoma |
| 34 | 74 | Female | Sphenoid crest | ICA | YES | YES | Meningioma |
| 35 | 24 | Male | Optic canal | ICA | YES | YES | NA |
| 36 | 40 | Male | Neck | MCA、ICA、ECA | YES | YES | Schwannoma |
| 37 | 59 | Male | Sphenoid crest | ICA、MCA、ACA | YES | YES | Meningioma |
| 38 | 34 | Female | Frontal lobe | cavernous malformation | YES | YES | Vascular Malformation |
| 39 | 45 | Female | Cavernous sinus | ICA | YES | YES | Meningioma |
| 40 | 57 | Female | paranasal sinuse | ICA | NO | YES | Schwannoma |
| 41 | 73 | Female | CPA | VA | YES | YES | Meningioma |
| 42 | 56 | Male | sella region | ICA | YES | YES | Chordoma |
| 43 | 52 | Female | Cavernous sinus | ICA | YES | YES | Meningioma |
| 44 | 66 | Female | sella region | ICA | YES | YES | Pituitary Adenoma |
| 45 | 59 | Female | sella region | ICA | YES | YES | Pituitary Adenoma |
| 46 | 65 | Female | Sphenoid crest | ICA、MCA、ACA | YES | YES | Meningioma |
| 47 | 41 | Female | Neck | MCA | YES | YES | Schwannoma |
| 48 | 26 | Male | Neck | VA | NO | YES | Schwannoma |
| 49 | 66 | Female | sella region | ICA | YES | YES | Chordoma |
| 50 | 69 | Female | Parasellar | ICA | YES | YES | Cyst |
| 51 | 63 | Female | sella region | ICA | YES | YES | Pituitary Adenoma |
| 52 | 62 | Female | Parasellar | ICA | YES | YES | Meningioma |
| 53 | 54 | Male | sella region | ICA | NO | YES | Pituitary Adenoma |
| 54 | 52 | Male | Cavernous sinus | ICA | YES | YES | Schwannoma |
| 55 | 45 | Female | Infratemporal fossa | ICA | NO | YES | Anaplastic Meningioma |
| 56 | 33 | Female | Cavernous sinus | ICA | YES | YES | Chondroma |

Abbreviations: ICA, internal carotid artery; MCA, middle cerebral artery; ACA, anterior cerebral artery; PCA, posterior cerebral artery; VA, vertebral artery; ECA, external carotid artery; MVD, microvascular Doppler; A/V, artery or vein involved.
