# Supplementary material for: Complex skull base brain tumor resection: the role of microvascular doppler in surgical precision and outcomes
Source: Front Oncol. 2025 Sep 3;15:1600980. doi: 10.3389/fonc.2025.1600980 (PMC12441036; doi:10.3389/fonc.2025.1600980)
Supplement: Supplementary file 3 [file Table2.docx]

Supplementary Table 2. Raw grayscale measurements and computed CNR values for intraoperative imaging across five representative cases.

| Case | Stage | μ_v (vessel) | μ_b (background) | σ_b (background) | CNR |
| --- | --- | --- | --- | --- | --- |
| 24 | Before MVD | 77.791 | 70.185 | 9.265 | 0.821 |
|  | After MVD | 98.169 | 71.601 | 10.291 | 2.582 |
| 41 | Before MVD | 120.04 | 114.203 | 13.059 | 0.447 |
|  | After MVD | 136.217 | 82.277 | 15.659 | 3.445 |
| 55 | Before MVD | 220.958 | 179.325 | 28.131 | 1.480 |
|  | After MVD | 232.637 | 176.048 | 22.227 | 2.546 |
| 56 | Before MVD | 143.13 | 135.43 | 17.314 | 0.445 |
|  | MVD | 174.314 | 121.423 | 18.344 | 2.883 |
|  | ICG | 194.134 | 125.412 | 18.431 | 3.729 |
| 37 | Before MVD | 144.123 | 132.123 | 10.313 | 1.164 |
|  | MVD | 159.42 | 134.131 | 9.314 | 2.715 |
|  | ICG | 176.314 | 131.341 | 11.312 | 3.976 |

This table summarizes the grayscale intensity values and resulting contrast-to-noise ratio (CNR) for five representative patients. Measurements were taken at three imaging stages: before MVD, after MVD, and after FLOW800 (ICG) imaging when available. Vessel and background regions of interest (ROIs) were manually selected on intraoperative grayscale-converted images to calculate CNR. These data support the visual enhancement observed with MVD and dual-modality imaging in skull base tumor resections.
Abbreviations: μ_v: Mean grayscale intensity of the vessel ROI; μ_b: Mean grayscale intensity of the background ROI; σ_b: Standard deviation of background ROI grayscale intensity; CNR: Contrast-to-noise ratio
